# Production of Quorum Sensing Inhibitors in Growing Onion Bulbs Infected with *Pseudomonas aeruginosa* E (HQ324110)

**DOI:** 10.5402/2012/161890

**Published:** 2012-01-05

**Authors:** Mohamed H. Abd-Alla, Shymaa R. Bashandy

**Affiliations:** Botany Department, Faculty of Science, Assiut University, Assuit 71516, Egypt

## Abstract

Eighteen organic compounds were present in growing onion bulbs cultivar Giza 6 infected with *P. aeruginosa*, but only fourteen of them are present in dry infected onion bulbs; however, four compounds were missing in dry onion. The missing compounds in dry infected onion bulbs are pantolactone, 4,5-dihydro-4,5-dimethylfuran-2(3H)-one, myristic acid, and linoleic acid. All of them were detected in growing onion (living cell) during *Pseudomonas aeruginosa* infection, and it is hypothesized that it may be produced by plants and act as defence system. Pantolactone and myristic acid were selected to explore their effects on growth and virulence factors of *Pseudomonas aeruginosa*. Exogenous application of pantolactone and myristic acid significantly inhibited pyocyanin production, protease, and lipase and polygalacturonase activity but did not have any significant effects on bacterial growth. The inhibition of virulence factors without reduction in bacterial growth may be providing strong support that these chemical molecules are general quorum sensing inhibitors than an antibacterial effect. Disruption of quorum sensing of pathogen indicates that this new approach has potential in fighting bacterial infections in human and plants.

## 1. Introduction

*Pseudomonas aeruginosa* is an opportunistic pathogen capable of eliciting soft-rot symptoms when infiltrated into a variety of plants including tomato, lettuce, onion, and tobacco [1–5]. *P. aeruginosa *is also known to be a common colonizer of many fruits and green plants and can persist without causing disease symptoms [6]. Virulence of *P. aeruginosa* is multifactorial, involving both secreted and cell-associated bacterial products, such as proteases, lipase, polygalacturonase, and pyocyanin [7]. Expression of these virulence factors appears to be controlled by signal molecule-dependent cell-cell communication systems, which are used by *P. aeruginosa* to monitor its own population density in a process known as quorum-sensing and global virulence regulation networks [8].

Plants have evolved multiple defense strategies for combating invading pathogens. Plant defence, including physical defence based on, for example, cuticle structure, various thorns and hairs, and chemical defence based on synthesis of chemical compounds can be divided into constitutive and inducible defence components [9, 10]. Constitutive defence can be found in healthy plants that are not harmed by biotic or abiotic stressors. In many circumstances, constitutive defence is not enough to protect against pathogen [11, 12].

Microbial infection will lead to form or increase some new protective plant structures or production of new chemical compounds in plant tissues. Tissue concentration and volatile emissions of organic compounds may both be elevated, but responsiveness of different compounds depends on the type of attacking organism [12].

Many eukaryotic organisms are able to produce and secrete compounds that mimic the quorum sensing signals of bacteria and thus affect the behavior of associated bacteria [13, 14]. The halogenated furanones of *Delisea pulchra*, a marine red alga, were observed to share structural similarity with bacterial acyl homoserine lactone and were shown to strongly and specifically inhibit quorum-sensing-regulated behaviors in a variety of bacterial species [15]. Higher plants, such as pea, crown vetch, *M. truncatula*, rice, soybean, and tomato, were also shown to produce substances that appear to mimic the activities of acyl homoserine lactone and have specific effects on quorum-sensing-regulated behaviors in bacteria [15–17].

The present work aimed to identify some chemical molecules induced in growing onion infected with *P. aeruginosa*. Also, the effect of exogenous application of pantolactone and myristic acid on the growth and production of virulence factors of *P. aeruginosa* in vitro has been addressed. Chemical molecules that target quorum sensing have been proposed as an antivirulence strategy that could be used in control of bacterial disease.

## 2. Material and Method

### 2.1. Bacterial Strains

*Pseudomonas aeruginosa *isolate E (HQ324110) was recovered from onion bulbs (*Allium cepa*) collected from vegetable markets of Assuit governorate, Egypt, and was used in this study.* Pseudomonas aeruginosa* isolate E was identified based on phenotypic characteristics and by sequencing of the 16S rRNA gene [4].

### 2.2. Determination of Some Chemical Compounds in Infected and Uninfected Onion Bulbs

*Pseudomonas aeruginosa* E (HQ324110) was inoculated into growing (cultivated) onion bulbs cv Giza 6 and dry bulb storage onions cv Giza 6. With the use of a 0.25 G syringe needle, wounds were made on an onion bulb to inoculate the causal agent. The bacterial inocula were obtained from 2-day-old cultures on KB broth medium incubated at 30°C. The bacterial causal agent was inoculated longitudinally from the neck part and transversely from the outer to the inner part of the onion bulb at the level of 5 mL of *Pseudomonas aeruginosa *(10^8^ cell mL) on wound. Inoculated onion bulbs were incubated at 30°C in a moist chamber. Inoculated bulbs were examined daily for development of symptoms. Uninfected growing and dry onion bulbs were used as control. Three days after infection, 1 g of plant tissue was grounded in 2 equal volumes of ethyl acetate containing 0.01% (final concentration) glacial acetic acid using mortar. The ethyl acetate phase was collected and stored at 20°C. Samples of extracts were dried under a stream of sterile air, and each residue was redissolved in 50% methanol and analyzed by Gas chromatography-mass spectrometry (GC/MS Model 6890N/5975B Agilent Technology). The chromatograph was equipped with DB-Wax capillary column. The flow rate of helium (carrier gas) was 1.5 mL/min. The injection volume was 1 *μ*L; injector temperature was programmed from 20 to 250°C at 180°/min then held at 250°C for 80 min. The oven temperature was at 60°C for 3 min, and from 60 to 220°C at 2°C/min, from 220°C to 245°C at 3°C/min then held 20 min at 245°C. The flame ionization detector temperature was 250°C. Mass spectra (MS) were recorded in the electronic impact (EI) and positive chemical ionization (PCI) modes. The transfer line temperature was 250°C with source temperature of 250°C. Mass spectra were scanned at 70 eV (EIMS) and 230 eV (PCIMS) in the range m/e 29–350 amu at 1 s intervals [18, 19]. Identification of the components was done on the basis of retention index and the comparison of EI mass spectra with reference compounds.

### 2.3. Effect of Exogenous Application of Pantolactone and Myristic Acid on Bacterial Growth

Pantolactone and myristic acid (Alfa Aesar, USA) were added at 25, 50, and 100 *μ*M to King's B medium [20]. Flasks containing 25 mL of medium were inoculated with 1 mL of *Pseudomonas aeruginosa *(10^8^ cell mL) and incubated on an orbital shaker at 150 rpm/min at 30°C. The growth response of bacterial strains was assessed by following the optical density at 540 nm and was compared by control. Bacterial growth was measured over the period from hours 2 to 72.

### 2.4. Effect of Pantolactone and Myristic Acid on Pyocyanin Production by *Pseudomonas aeruginosa*

Pantolactone and myristic acid were added at 25, 50, and 100 *μ*M to King's B medium. Cultures were incubated in an orbital shaking incubator for 9.5 h at 150 rpm and 30°C. Pyocyanin was extracted in 1 mL chloroform, followed by a second extraction into 150 *μ*M 0.2 N HCl. The culture broth was then centrifuged at 8000 rpm to remove cells. The absorbance of this solution was measured at 520 nm [21]. Each treatment was conducted with three replicates.

### 2.5. Effect of Pantolactone and Myristic Acid on Protease, Lipase, and Polygalacturonase Production by *Pseudomonas aeruginosa*

*Pseudomonas aeruginosa* isolate E (HQ324110) was grown in 50 mL of liquid medium in an Erlenmeyer flask (250 mL) containing (g/L): MgSO_4_·7H_2_O 0.2, K_2_HPO_4_ 2.0, KH_2_PO_4_ 2, and casein 10 (pH 8) [22]. The growth medium was supplemented with different concentration of pantolactone and myristic acid (0, 25, 50, and 100 *μ*M). Assays for proteolytic activity were performed according to Ohara-Nemoto et al. [23]. One unit of enzyme activity was defined as the amount of enzyme that releases 1 mg mL^−1^ tyrosine per min under the assay conditions. Controls contained autoclaved enzymes instead of active enzymes that were used. For lipase assay, the basal medium consisted of (g/L): bacteriological peptone 15.0, yeast extract 5.0, NaCl 2.0, MgSO_4_ 0.4, K_2_HPO_4_ 0.3, KH_2_PO_4_ 0.3, and olive oil 10.0 mL for lipase induction [24]. The medium was amended with different concentration of pantolactone and myristic acid. Lipase activity was measured by universal titrimetric method [25]. The activity was measured as amount of enzyme required liberating 1 *μ*M mL^ −1^ fatty acid per min. The basal medium for pectinase production consisted of (g/L): pectin 4, yeast extract 2, NH_4_Cl 1, MgSO_4_ 0.5 [26]. Pantolactone, and myristic acid were added at concentration of 0, 25, 50, and 100 *μ*M to the growth medium. Cultures were incubated in an orbital shaking incubator for 36 h at 150 rpm and 37°C. The culture broth was then centrifuged at 8000 rpm to remove cells. The clear supernatant was collected for enzymes assay. The hydrolytic pectic enzymes were determined according to method of Gomes et al. [26]. A unit of activity is defined as that amount of enzyme which catalyzes the release of 1 mmol of reducing groups per minute and expressed as U/mL. Each treatment was conducted with three replicates.

### 2.6. Protein Measurement

Cellular protein was determined by the method of Lowry et al. [27] and with bovine serum albumin as the standard.

### 2.7. Statistical Analysis

Experimental data were subject to a one-way analysis of variance using a computer program (PC-state). Means were compared to test significance between treatments using the LSD at 5% probability [28].

## 3. Results and Discussion

### 3.1. Identification of Some Chemical Compounds Produced in an Infected and Uninfected Onion Bulbs

To explore the role of bacterial infection on the induction of chemical compounds in onion tissues, ethyl acetate extracts of artificially infected dry onion and spring onion tissues were analyzed by GC/MS and compared with the uninfected onion tissues at 2 days after infection (Table 1). Based on the extraction method, the compounds obtained in infected growing onion tissues were identified as 4,5-dihydro-4,5-dimethylfuran-2(3H)-one, octanal, 5-methyl furfural, 2-dodecene, pantolactone, undecane, 2-decenoic acid, 2-hydroxy decenoic acid, methyl 2,3,5-tris-O-methyl-4-thio alph, D-arabinofuranoside, myristic acid, (3.alpha.), cholest-5-en-3-ol, palmitic acid, linoleic acid, 3-dihydro-2(3H) furanone, 1-heptadecene, 1-octadecene, canonical (2-ethoxydecylphthalimide), and octacosane. However, we found four compounds (i.e., as pantolactone, 4,5-dihydro-4,5-dimethylfuran-2(3H)-one, myristic acid, and linoleic acid) missing in infected storage onion tissues as compared with infected growing onion tissue.

Some of these compounds might be produced by plant or by bacteria. The diffusible signal factor (DSF) was recently identified as unsaturated fatty acids which are produced by a variety of of phytopathogenic bacteria. Wang et al. [29] identified the DSF in *Xanthomonas campestris*, as cis-11-methyl-2-dodecenoic acid which is an *α*, *β*-unsaturated fatty acid which regulates polysaccharide and extracellular enzyme production both virulence factors. They reported that several other pathogenic bacteria such as *P. aeruginosa *and *Mycobacterium* sp. also display diffusible signal factor like activity. Yim et al. [30] have argued that the majority of low-molecular-weight organic compounds made and secreted by microbes are likely to function as cell-signalling molecules which modulate the metabolic activities of natural microbial communities.

The discovery that the red alga *Delisea* produces furanone inhibitors of bacterial quorum sensing stimulated a search for similar activities in plants [31]. This approach is greatly attractive because it does not impose harsh selective pressure for the development of resistance as with antibiotics, because quorum sensing is not directly involved in processes essential for growth of bacteria. The halogenated furanones from *D. pulchra* provided the first example of quorum sensing compounds produced by a eukaryote [31, 32]. It was recently demonstrated that several plants, including pea seedlings [33], garlic [34], *Medigo sativa* [35, 36], vanilla [37], *Scorzonera sandrasica* [38], and *Tremella fuciformis *extract [39], are also able to produce compounds that interfere with bacterial QS [40].

This implies that the quorum sensing signal-mimic compounds are produced by plants to help them deal with the diversity of bacteria that they encounter, just as the furanone mimics of *D. pulchra* help the alga to control the colonization and fouling of its surfaces.

### 3.2. Inhibition of Virulence Factor Production by Pantolactone and Myristic Acid

**Figure 1:**
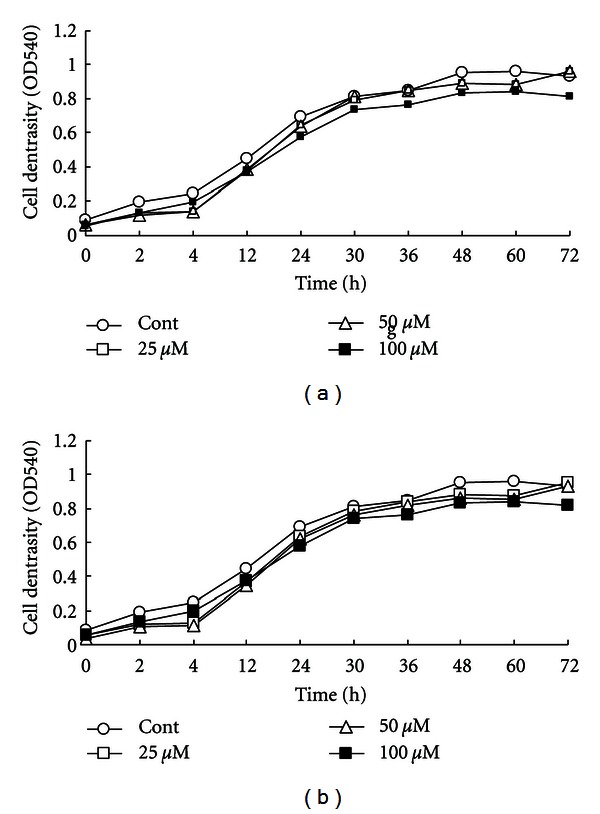
Effect of pantolactone (a) and myristic acid (b) on the growth rate of *Pseudomonas aeruginosa*. Values represent the mean of three replicates for each treatment.

An experiment was designed to investigate the effect of different level of myristic acid and pantolactone on growth and production of extracellular virulence factors. Data presented in Figure 1 show that both tested substances did not have any significant effects on bacterial growth as measured by optical density at 540 nm. To confirm that pantolactone and myristic acid act as quorum sensing inhibitors and effect on virulence factors, we measured the levels of four extracellular virulence factors, namely, pyocyanin, protease, lipase, and polygalacturonase in *Pseudomonas aeruginosa* culture fluid.

**Figure 2:**
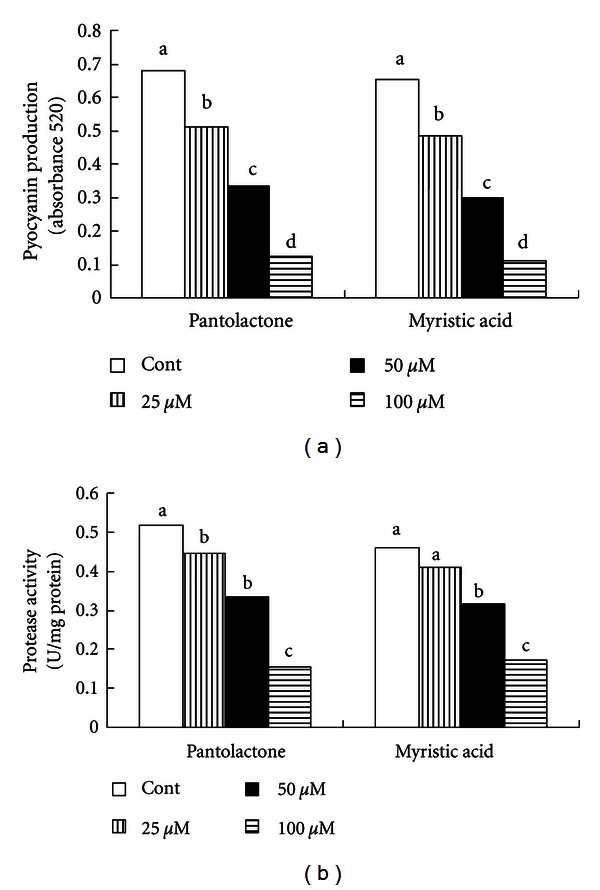
Effect of pantolactone and myristic acid on pyocyanin production (a) and protease production (b) by* Pseudomonas aeruginosa*. Values represent mean of three replicates for each treatment. Means with the same letter are not significantly different between treatments within a compound at the 5% level, using an LSD test.

**Figure 3:**
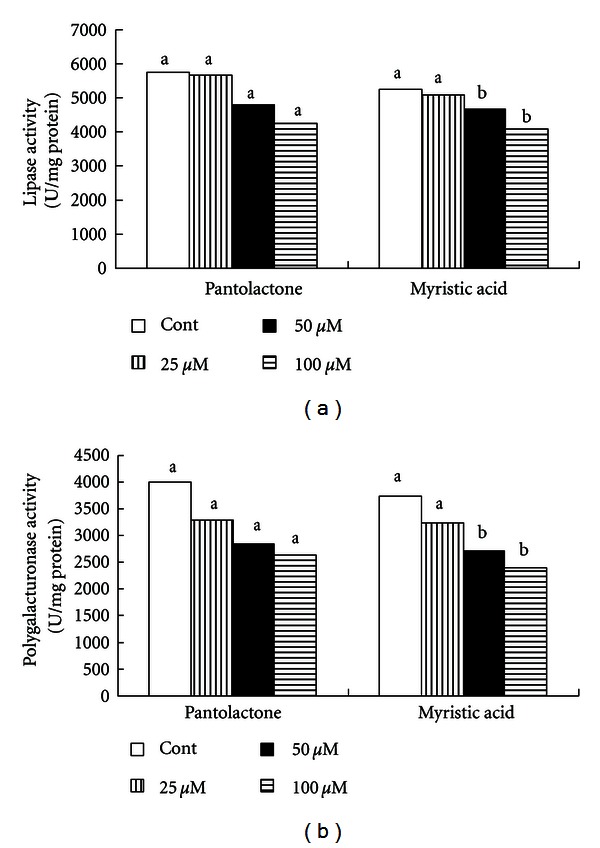
Effect of exogenous application of pantolactone and myristic acid on lipase production (a) and polygalacturonase production (b) by* Pseudomonas aeruginosa*. Values represent mean of three replicates for each treatment. Means with the same letter are not significantly different between treatments within a compound at the 5% level, using an LSD test.

 It is obvious that pyocyanin formation by *Pseudomonas aeruginosa* was reduced about 25% in the presence of 25 *μ*M of pantolactone and about 65% in the presence of 50 *μ*M of pantolactone (Figure 2(a)). Similarly to pantolactone, the addition of 25 *μ*M of myristic acid to growth resulted in a 26% reduction in pyocyanin medium pyocyanin formation (Figure 2(a)). However, 50 *μ*M of myristic acid resulted in a 55% decrease in pyocyanin formation. The production of protease, lipase, and polygalacturonase was significantly inhibited in the presence of myristic acid (Figures 2(b) and 3). Similarly, pantolactone had the same effect except for lipase production (Figures 2(b) and 3).

It is clear that these compounds inhibited some virulence factors (pyocyanin, protease, lipase, and polygalacturonase production) without reduction in bacterial growth. This effect may be providing strong support that these chemical molecules may act as quorum sensing inhibitors rather than an antibacterial effect (quorum-quenching mechanisms are unrelated to static or cidal effects). Data of current research indicate that myristic acid and pantolactone may act as negative signals for regulation of bacterial virulence. Our finding supports the work of Liaw et al. [41] who found that some saturated fatty acids inhibited swarming and virulence factor expression in *Proteus mirabilis*, *Serratia marcescens*, and *Salmonella enterica*. Hentzer et al. [42] demonstrated that the *Pseudomonas aeruginosa* communication systems can be blocked by halogenated furanone compound. This is a highly specific and effective approach to attenuating bacterial virulence and controlling bacterial infections [43, 44]. Wu et al. [45] showed that synthetic furanone compound inhibited bacterial quorum sensing and simultaneously reduced the severity of the disease. Defoirdt et al. [46] found that the natural furanone (5Z)-4-bromo-5-(bromomethylene)-3-butyl-2(5H)-furanone disrupts quorum-sensing-regulated gene expression in *V. harveyi* by decreasing the DNA-binding activity of the quorum sensing master regulator protein. A group of researchers has collaborated and continuously studied the inhibition of QS using the *P. aeruginosa* model. They have proved the inhibitory activity of furanone compounds [42], South Florida plant extract [47], and garlic extract [34] in *P. aeruginosa*.

In plant pathology, there is major interest to find natural or synthetic compounds active in small quantities that are capable of interfering with quorum sensing in pathogenic bacteria in order to disrupt their pathogenicity/virulence factor production.

Bacterial diseases are much more difficult to control than fungal diseases because of the lack of effective and benign plant protection products. Using quorum sensing as a target to control and handle detrimental infections caused by human, animal, and plant pathogens is potentially an attractive strategy.

## Figures and Tables

**Table 1:** Chemical compounds produced in onion bulbs infected with *Pseudomonas aeruginosa. *Uninfected onion bulbs were used as control.

Chemical compounds	Retention time	Growing onion		Dry onion	
		Uninfected	Infected	Uninfected	Infected
4,5-Dihydro-4,5-dimethylfuran-2(3H)-one	12.69	−	+	−	+
Octanal	14.205	−	+	−	+
5, Methyl furfural	14.692	−	+	−	+
2-dodecene	16.301	−	+	−	+
pantolactone	16.449	−	+	−	−
Undecane	17.282	−	+	−	+
2-decenoic acid	21.640	−	+	−	+
2-hydroxy, decenoic acid	24.447	−	+	−	+
Methyl 2,3,5-tris –O-methyl-4-thio alph. D-arabinofuranoside	25.7	−	+	−	+
Myristic acid	27.2	−	+	−	−
(3.alpha.),cholest-5-en-3-ol	28.5	−	+	−	+
Palmitic acid	29.8	−	+	−	+
linoleic acid	33.4	−	+	−	−
3-dihydro-2(3H)furanone	33.6	−	+	−	−
1-Heptadecene	35.6	−	+	−	+
1-Octadecene	35.7	−	+	−	+
Canonical (2-Ethoxydecylphthalimide)	39.9	−	+	−	+
Octacosane	40.4	−	+	−	+

## References

[1] Kominos SD, Copeland CE, Grosiak B, Postic B (1972). Introduction of *Pseudomonas aeruginosa* into a hospital via vegetables. *Applied Microbiology*.

[2] Abd-Alla MH, Bashandy SR (2008). Bacterial wilt and spot of tomato caused by *Xanthomonas vesicatoria* and *Ralstonia solanacearum* in Egypt. *World Journal of Microbiology & Biotechnology*.

[3] Abd-Alla MH, Bashandy SR, Schnell S (2010). Occurrence of xanthomonas axonopodis pv. phaseoli, the causal agent of common bacterial blight disease, on seeds of common bean (Phaseolus vulgaris L.) in upper Egypt. *Folia Microbiologica*.

[4] Abd-Alla MH, Bashandy SR, Ratering S, Shnell S (2011). First report of soft rot of onion bulbs in storage caused by *Pseudomonas aeruginosa* in Egypt. *Journal of Plant Interaction*.

[5] Abd-Alla MH, Bashandy SR, Schnell S, Ratering S (2011). Isolation and characterization of Serratia rubidaea from dark brown spots of tomato fruits. *Phytoparasitica*.

[6] Cho JJ, Schroth MN, Kominos SD, Green SK (1975). Ornamental plants as carriers of *Pseudomonas aeruginosa*. *Phytopathology*.

[7] Rahme LG, Stevens EJ, Wolfort SF, Shao J, Tompkins RG, Ausubel FM (1995). Common virulence factors for bacterial pathogenicity in plants and animals. *Science*.

[8] Mole BM, Baltrus DA, Dangl JL, Grant SR (2007). Global virulence regulation networks in phytopathogenic bacteria. *Trends in Microbiology*.

[9] Karban R, Baldwin IT (1997). *Induced Responses to Herbivory*.

[10] Fray RG (2002). Altering plant-microbe interaction through artificially manipulating bacterial quorum sensing. *Annals of Botany*.

[11] Von Bodman SB, Bauer WD, Coplin DL (2003). Quorum sensing in plant-pathogenic bacteria. *Annual Review of Phytopathology*.

[12] Holopainen JK, Heijari J, Nerg A-M, Vuorinen M, Kainulainen P (2009). Potential for the use of exogenous chemical elicitors in disease and insect pest management of conifer seedling production. *The Open Forest Science Journal*.

[13] Bauer WD, Teplitski M (2001). Can plants manipulate bacterial quorum sensing?. *Australian Journal of Plant Physiology*.

[14] Kjelleberg S, Steinberg P, Lindow SE, Poinar E, Elliott V (2002). Defenses against bacterial colonization of marine plants. *Phyllosphere Microbiology*.

[15] Daniels R, De Vos DE, Desair J (2002). The cin quorum sensing locus of Rhizobium etli CNPAF512 affects growth and symbiotic nitrogen fixation. *Journal of Biological Chemistry*.

[16] Teplitski M, Robinson JB, Bauer WD (2000). Plants secrete substances that mimic bacterial N-acyl homoserine lactone signal activities and affect population density-dependent behaviors in associated bacteria. *Molecular Plant-Microbe Interactions*.

[17] Gao M, Teplitski M, Robinson JB, Bauer WD (2003). Production of substances by *Medicago truncatula* that affect bacterial quorum sensing. *Molecular Plant-Microbe Interactions*.

[18] Bureau SM, Razungles AJ, Baumes RL (2000). The aroma of Muscat of Frontignan grapes: effect of the light environment of vine or bunch on volatiles and glycoconjugates. *Journal of the Science of Food and Agriculture*.

[19] Schneider R, Baumes R, Bayonove C, Razungles A (1998). Volatile compounds involved in the aroma of sweet fortified wines (vins doux naturels) from Grenache noir. *Journal of Agricultural and Food Chemistry*.

[20] King EO, Ward MK, Raney DE (1954). Two simple media for the demonstration of pyocyanin and fluorescin. *The Journal of Laboratory and Clinical Medicine*.

[21] Essar DW, Eberly L, Hadero A, Crawford IP (1990). Identification and characterization of genes for a second anthranilate synthase in *Pseudomonas aeruginosa*: interchangeability of the two anthranilate synthase and evolutionary implications. *Journal of Bacteriology*.

[22] Chakraborty R, Srinivasan M (1993). Production of a thermostable alkaline protease by a new Pseudomonas sp. by solid substrate fermentation. *Journal of Microbiology and Biotechnology*.

[23] Ohara-Nemoto Y, Sasaki M, Kaneko M, Nemoto T, Ota M (1994). Cysteine protease activity of streptococcal exotoxin B. *Canadian Journal of Microbiology*.

[24] Baharum SN, Salleh AB, Razak CNA, Basri M, Rahman MBA, Rahman RNZRA (2003). Organic solvent tolerant lipase by *Pseudomonas* sp. strain S5: stability of enzyme in organic solvent and physical factors affecting its production. *Annals of Microbiology*.

[25] Fadiloğlu S, Söylemez Z (1997). Kinetics of lipase-catalyzed hydrolysis of olive oil. *Food Research International*.

[26] Gomes I, Saha RK, Mohiuddin G, Hoq MM (1992). Isolation and characterization of a cellulase-free pectinolytic and hemicellulolytic thermophilic fungus. *World Journal of Microbiology &amp; Biotechnology*.

[27] Lowry OH, Rosebrough NJ, Farr AL, Randall RJ (1951). Protein measurement with the Folin phenol reagent. *The Journal of Biological Chemistry*.

[28] Rao M, Blane U, Zonnenberg M (1985). *PC-State Version I A*.

[29] Wang LH, He Y, Gao Y (2004). A bacterial cell-cell communication signal with cross-kingdom structural analogues. *Molecular Microbiology*.

[30] Yim G, Huimi Wang H, Davies J (2006). The truth about antibiotics. *International Journal of Medical Microbiology*.

[31] Givskov M, De Nys R, Manefield M (1996). Eukaryotic interference with homoserine lactone-mediated prokaryotic signalling. *Journal of Bacteriology*.

[32] Manefield M, de Nys R, Kumar N (1999). Evidence that halogenated furanones from *Delisea pulchra* inhibit acylated homoserine lactone (AHL)-mediated gene expression by displacing the AHL signal from its receptor protein. *Microbiology*.

[33] Delalande L, Faure D, Raffoux A (2005). N-hexanoyl-L-homoserine lactone, a mediator of bacterial quorum-sensing regulation, exhibits plant-dependent stability and may be inactivated by germinating Lotus corniculatus seedlings. *FEMS Microbiology Ecology*.

[34] Rasmussen TB, Bjarnsholt T, Skindersoe ME (2005). Screening for quorum-sensing inhibitors (QSI) by use of a novel genetic system, the QSI selector. *Journal of Bacteriology*.

[35] Keshavan ND, Chowdhary PK, Haines DC, González JE (2005). L-canavanine made by Medicago sativa interferes with quorum sensing in *Sinorhizobium meliloti*. *Journal of Bacteriology*.

[36] Bauer WD, Mathesius U, Teplitski M (2005). Eukaryotes deal with bacterial quorum sensing. *ASM News*.

[37] Choo JH, Rukayadi Y, Hwang JK (2006). Inhibition of bacterial quorum sensing by vanilla extract. *Letters in Applied Microbiology*.

[38] Bosgelmez-Tinaz G, Ulusoy S, Ugur A, Ceylan O (2007). Inhibition of quorum sensing-regulated behaviors by *Scorzonera sandrasica*. *Current Microbiology*.

[39] Zhu H, Sun SJ (2008). Inhibition of bacterial quorum sensing-regulated behaviors by Tremella fuciformis extract. *Current Microbiology*.

[40] Bauer WD, Robinson JB (2002). Disruption of bacterial quorum sensing by other organisms. *Current Opinion in Biotechnology*.

[41] Liaw SJ, Lai HC, Wang WB (2004). Modulation of swarming and virulence by fatty acids through the RsbA protein in Proteus mirabilis. *Infection & Immunity*.

[42] Hentzer M, Wu H, Andersen JB (2003). Attenuation of *Pseudomonas aeruginosa* virulence by quorum sensing inhibitors. *The EMBO Journal*.

[43] Müh U, Schuster M, Heim R, Singh A, Olson ER, Greenberg EP (2006). Novel *Pseudomonas aeruginosa* quorum-sensing inhibitors identified in an ultra-high-throughput screen. *Antimicrobial Agents & Chemotherapy*.

[44] Ren D, Givskov M, Rasmussen TB, Balaban N, Balaban N (2008). Quorum-sensing inhibitory compounds. *Control of Biofilm Infections by Signal Manipulation*.

[45] Wu H, Song Z, Hentzer M (2004). Synthetic furanones inhibit quorum-sensing and enhance bacterial clearance in *Pseudomonas aeruginosa* lung infection in mice. *Journal of Antimicrobial Chemotherapy*.

[46] Defoirdt T, Miyamoto CM, Wood TK (2007). The natural furanone (5Z)-4-bromo-5-(bromomethylene)-3-butyl-2(5H)-furanone disrupts quorum sensing-regulated gene expression in *Vibrio harveyi* by decreasing the DNA-binding activity of the transcriptional regulator protein luxR. *Environmental Microbiology*.

[47] Adonizio A, Kong KF, Mathee K (2008). Inhibition of quorum sensing-controlled virulence factor production in *Pseudomonas aeruginosa* by south Florida plant extracts. *Antimicrobial Agents & Chemotherapy*.

